# Associations between genetic variants of the *POU1F1* gene and production traits in Saanen goats

**DOI:** 10.5194/aab-62-249-2019

**Published:** 2019-05-03

**Authors:** Raziye Işık, Güldehen Bilgen

**Affiliations:** 1 Faculty of Agriculture, Department of Agricultural Biotechnology, Tekirdağ Namık Kemal University, Tekirdağ, Turkey; 2 Faculty of Agriculture, Department of Animal Science, Ege University, İzmir, Turkey

## Abstract

This study was conducted to determine the polymorphisms of the *POU1F1* gene and their relationships with milk yield and components, litter size, birth weight, and weaning weight in goats. For this purpose, a total of 108 Saanen goats from two different farms (Bornova and Manisa) were used as animal materials. Polymorphisms at the exon 6 and the 3${}^{\prime }$ flanking region of the *POU1F1* gene were determined by using PCR-RFLP with *Pst*I and *Alu*I restriction enzymes and DNA sequencing analyses. Two alleles and three genotypes were identified by *Alu*I or *Pst*I digestions of the *POU1F1* gene. The genotypes frequencies of TT, TC, and CC were 64.8 %, 31.5 % and 3.7 % for the *Pst*I locus; 54.6 %, 31.5 % and 13.9 % for the *Alu*I locus, respectively. T allele frequencies (0.56 and 0.88 for the *Alu*I locus, 0.80 and 0.81 for the *Pst*I locus, respectively) were predominant in both loci at the Bornova and Manisa farms. In terms of *POU1F1*-*Alu*I and *POU1F1*-*Pst*I loci, two populations were found to be in Hardy–Weinberg equilibrium. In the *POU1F1*-*Alu*I locus, significant associations were found between genotypes and lactation milk yield and litter size. Similarly, a significant relationship between genotypes and birth weight in the *POU1F1*-*Pst*I locus (p<0.05) was determined. The TC and CC genotypes were observed to be higher than the TT genotype for lactation milk yield and litter size at the *POU1F1*-*Alu*I locus. Birth weight was found to be higher in animals that have the CC genotype at the *POU1F1*-*Pst*I locus. In conclusion, the *POU1F1* gene can be used as a molecular marker for economic features like reproduction, growth, milk content and yield in Saanen goats.

## 1 Introduction

Goat populations have increased in recent years in spite of changes in agriculture and technological progress in the world. Demand for goat milk and its products has increased because of an increase in health-conscious consumers. Goat milk provides great advantages to human nutrition such as high digestibility, being antiallergenic, and it has short- and medium-chain length fatty acids (Park et al., 2007; Getaneh et al., 2016). Therefore, goat breeding has become popular, and the goat population in Turkey has increased over the last 5 years (FAO, 2016).

Goat breeding is carried out in village flocks, plateaus or nomadic flocks in Turkey. However, in recent years in western Anatolia some entrepreneurs have invested money in intensive farming that provides goat milk to dairy farms. The Saanen breed and its hybrids are usually raised in such intensive farm enterprises (Kaymakçı and Dellal, 2006; Kaymakçı and Taşkın, 2006). It is known that the goat population is high in Çanakkale and Balikesir, especially in İzmir, in the western Anatolia region (FAO, 2016).

Genes related to economic traits and possible effects on production traits have been investigated using various DNA markers. One of these markers is single nucleotide polymorphisms (SNPs) that can occur in the forms of transitions or transversions. SNP markers are used for identifying genetic diversity, in quantitative trait locus (QTL) analysis and in genomic selection of livestock. Investigations have been performed to determine SNPs affecting characteristics such as resistance to mastitis and scrapie diseases, carcass and meat quality, milk yield, and milk fat and protein content because SNPs are a widespread polymorphism found in the genome, and are easy to identify (Li et al., 2011; Zhang et al., 2012; Corral et al., 2013; Wang et al., 2015; dos Santos et al., 2015; Paiva et al., 2016; Ekegbu et al., 2019).

POU1F1 (also called GHF-1 or PIT-1) is a member of the POU-domain family which is an important regulator for growth hormone (GH), prolactin (PRL), and thyroid-stimulating hormone β (TSHβ) (Tuggle and Trenkle, 1996; Cohen et al., 1997; Li et al., 2016). Many transcription factors are involved in pituitary organogenesis during development and maturation of the anterior pituitary gland. POU homeodomains such as PROP1 and POU1F1, and PITX homeodomains such as PITX2 and PITX1 have been associated with a decrease in GH and PRL expression, and with proliferation of somatotropic and lactotropic cell lines (Savage et al 2003; Huai et al., 2011; Selvaggi and Dario, 2011). The *POU1F1* gene is located on 1q21–22 chromosome in goats, cattle and sheep and consists of 6 exons and 5 introns (Woollard et al., 2000).

The *POU1F1* gene is an important candidate gene associated with growth, reproduction, milk yield, and milk components. This is because the POU1F1 transcription factor regulates the expression of genes *GH*, *PRL*, and *TSH*β (Daga et al., 2013; Feng et al., 2012; Lan et al., 2009b, 2007a, b). Research has been carried out to investigate the association of the *POU1F1* gene with milk yield, milk composition, and growth traits in goats and cattle (Lan et al., 2007a, b; Zhang et al., 2009; Zhou et al., 2016). Some of the polymorphisms in the *POU1F1* gene were reported to be related to growth, weaning weight, litter size and meat quality traits in sheep (Mura et al., 2012; Özmen et al., 2013; Sadeghi et al., 2014; Jalil-Sarghale et al., 2014; Bai et al., 2016; AL-Khuzai and AL-Anbari 2018), milk production in cattle (Ahmadi et al., 2015), carcass weight in cattle (Seong et al., 2011), meat quality in rabbits (Wang et al., 2015), and milk production, growth traits and litter size in goats (Daga et al., 2013; Feng et al., 2012; Ma et al, 2017). Many studies have suggested that the *POU1F1* gene may be a candidate gene to be used in marker-assisted selection programs (Feng et al., 2012; Ma et al, 2017; AL-Khuzai and AL-Anbari 2018).

This study aimed to investigate the polymorphisms of the *POU1F1* gene and to evaluate their relationships with some characteristics of reproduction, growth, milk yield and milk components in Saanen goats that are reared in İzmir and Manisa Province.

## 2 Materials and methods

### 2.1 Samples and DNA isolation

A total of 108 Saanen goats (60 goats reared in the Small Ruminant Animal Application and Research Unit, Ege University Faculty of Agriculture Department of Animal Science, Bornova; 48 goats reared in a private Saanen farm in Manisa Province) and their offspring were used as materials. A total of 162 offspring from 108 dams were used for litter size, and 88 offspring from 60 dams were used for birth weight and weaning weight.

The monthly milk yield of individuals was recorded twice a day during lactation in 2013–2014. Protein, fat and dry matter ratios were determined in milk samples with a Bentley 150 milk analyzer. 10 mL of blood sample from the vena jugularis of the 108 goats was collected in vacuum tubes containing K3 EDTA as anticoagulant. Genomic DNA was isolated using a commercial DNA isolation kit (K0721, GeneJET Whole Blood Genomic DNA Purification Mini Kit, Thermo Fisher Scientific) according to the manufacturer's protocol.

### 2.2 DNA amplification and genotyping

The exon 6 and 3${}^{\prime }$ flanking region of *POU1F1* gene was amplified using F: 5${}^{\prime }$-CCATCATCTCCCTTCTT-3${}^{\prime }$ and R: 5${}^{\prime }$-AATGTACAATGTCCTTCTGAG-3)${}^{\prime }$ primers (Lan et al., 2007b). The 25 µL PCR volume contained 100 ng genomic DNA, 0.5 µM of each primers, 1× PCR Buffer, 200 µM dNTP, 2 mM ${\mathrm{MgCl}}_{2}$ and 1 U of Taq DNA polymerase (i-StarTaq^™^ DNA Polymerase, iNtRon Biotechnology). The cycling protocol was 5 min at 95 ${}^{\circ }$C, 35 cycles of 94 ${}^{\circ }$C for 30 s, 54 ${}^{\circ }$C annealing for 30 s, 72 ${}^{\circ }$C for 45 s with a final extension at 72 ${}^{\circ }$C for 10 min.

PCR products of the *POU1F1* gene were digested with 10 U of *Alu*I and *Pst*I restriction enzymes (FD0014 and FD0614, Thermo Fisher Scientific) at 37 ${}^{\circ }$C for 3 h. PCR products and restriction fragments were electrophoresed on a 2.5 % agarose gel stained with SafeView^™^ Classic (Applied Biological Materials Inc., Canada).

*POU1F1* gene fragments which gave different genotypes were also sequenced. The sequences of *POU1F1* fragments were analyzed by using the MEGA6 software (Molecular Evolutionary Genetics Analysis, version 6.0; Tamura et al., 2013) for generating sequence alignments.

### 2.3 Statistical analysis

The genotypic and allelic frequencies of the *POU1F1* gene and the Hardy–Weinberg equilibrium of the populations were calculated using the PopGene program (Yeh et al., 2000). The statistical software SPSS 18.0 was used to analyze the relationships between the genotypes and economic traits in goats.

Lactation milk yield was calculated according to the Trapeze II method, and the lactation period of 210 d was corrected to 280 d (ICAR, 2014). The total milk yield for each farm studied was statistically analyzed by the general linear model at significance level (α<0.05).

The adjusted *linear model I* with fixed effects was used to analyze the relationships between genotypes, milk yield, and components of 108 dairy goats. *linear model I*: $Y_{ijklm}=\mu +B_{i}+A_{j}+G_{k}+S_{l}+e_{ijklm}$, where $Y_{ijklm}$ was the milk traits measured of each *ijklm*th animal, μ the overall mean, $B_{i}$ the type of ith farm, $A_{j}$ the jth lactation number, $G_{k}$ the type of the kth genotype, $S_{l}$ the type of the lth birth, and $e_{ijklm}$ was the random error. The adjusted *linear model II* with fixed effects was used to analyze the relationships between genotype, birth weight, and weaning weight of 88 offspring. *Linear model II*: $Y_{ijkl}=\mu +S_{i}+G_{j}+C_{k}+b(X_{ijk}-X)+e_{ijkl}$, where $Y_{ijkl}$ was the weight traits measured on each of the ijklth animal, μ was the overall population mean, $S_{i}$ the type of birth of the ith offspring, $G_{j}$ the type of the jth genotype, $C_{k}$ the sex of the kth offspring (male, female), b the regression coefficient ($X_{ijk}$; birth weight of dam, X; overall birth weight population mean of dam for birth weight, $X_{ijk}$; weaning weight of offspring, X; overall weaning weight population mean of offspring for weaning weight), and $e_{ijkl}$ was the random error. Effects associated with the farm and season of birth were not into incorporated into the linear model because the preliminary statistical analyses indicated that these effects did not have significant influences on the variability of traits in populations. The adjusted *linear model III* with fixed effects was used to analyze the relationships between genotype and litter size of 162 offspring. *linear model III*: $Y_{ijkl}=\mu +K_{i}+A_{j}+G_{k}+e_{ijkl}$, where $Y_{ijkl}$ was the litter size trait measured for each $ijk_{l}$th animal, μ was the overall population mean, $K_{i}$ the type of ith farm, $A_{j}$ the jth lactation number, $G_{k}$ the type of the kth genotype, and $e_{ijkl}$ was the random error. Effects associated with the farm and season of birth were not incorporated into the linear model.

## 3 Results and discussion

**Figure 1:**
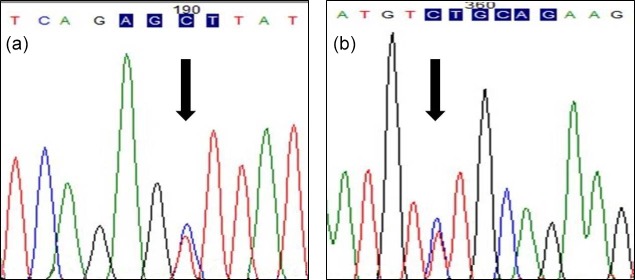
Sequence of *POU1F1* gene **(a)** *Alu*I restriction site **(b)** *Pst*I restriction site.

**Figure 2:**
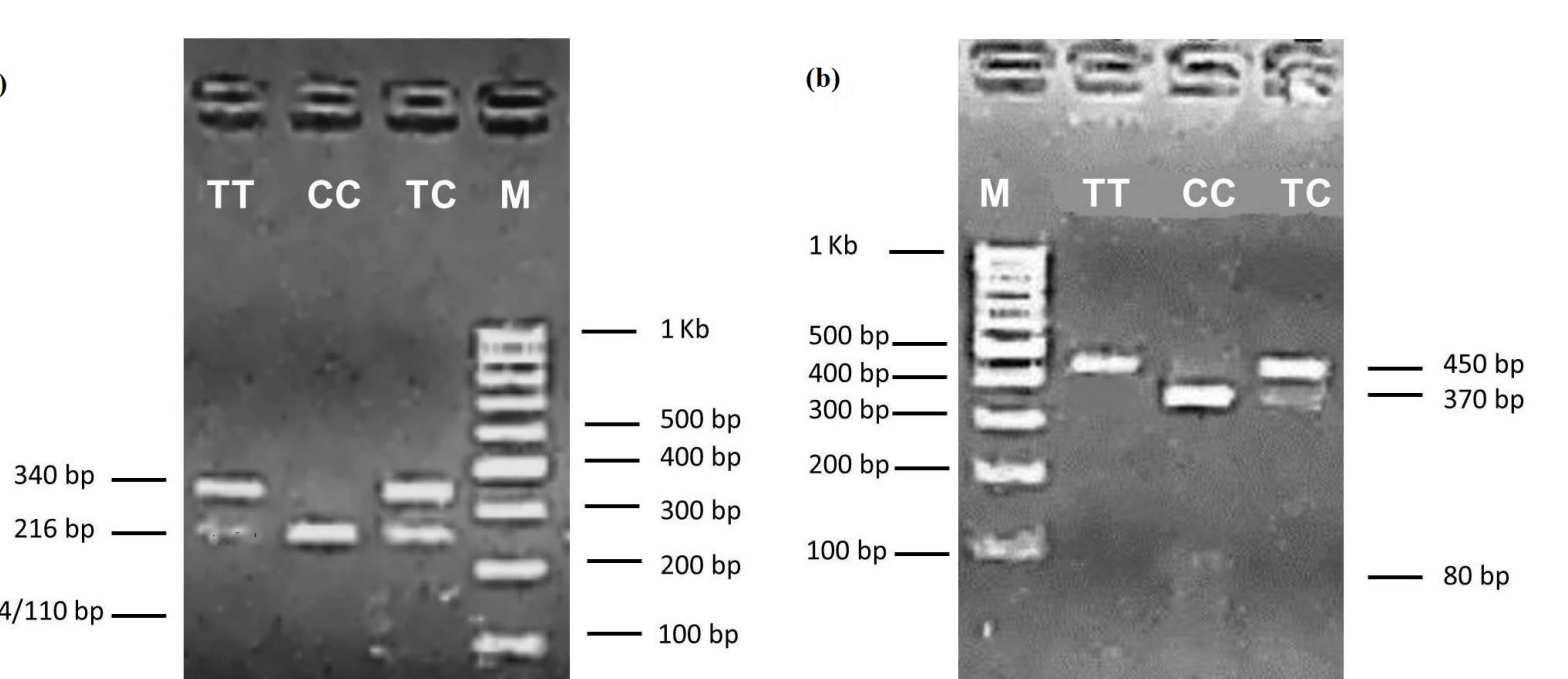
Electrophoresis patterns of the *POU1F1-Alu*I **(a)** and *POU1F1*-*Pst*I **(b)** loci. TT, TC and CC are genotypes for the *POU1F1-Alu*I and *POU1F1-Pst*I loci, M; marker.

PCR–restriction fragment length polymorphism (PCR–RFLP) with *Alu*I and *Pst*I restriction enzymes and DNA sequencing were used to validate genetic polymorphism for exon 6 to the 3${}^{\prime }$ flanking region of the *POU1F1* gene (450 bp) in two Saanen goat populations in Turkey. The transition from thymine to cytosine (DQ826413.1) (g.172T>C) in the sixth exon of the (210 bp long) *POU1F1* gene was determined by the restriction enzyme *Alu*I (Fig. 1a). The transition from cytosine to thymine (DQ826413.1) (g.110C>T) in the 3${}^{\prime }$ flanking region (total length 195 bp) was examined by the *Pst*I restriction enzyme (Fig. 1b). Two alleles and three genotypes were identified with *Alu*I (TT: 340, 110 bp; TC: 340, 216, 124, 110 bp; CC: 216, 124, 110 bp) and *Pst*I (TT: 450 bp; TC: 450, 370, 80 bp; CC: 370, 80 bp) loci of the *POU1F1* gene (Fig. 2a, b). The genotypes and allele frequencies of *POU1F1* gene-*Alu*I and *POU1F1* gene-*Pst*I analyses are listed in Table 1. *POU1F1*-T allele frequencies were 0.7 and 0.8 for *POU1F1-Alu*I and *POU1F1-Pst*I loci, respectively. The CC genotype was not found in Manisa farm for two loci. The *POU1F1* gene sequenced that is identified in this study was deposited to the NCBI GenBank with the accession number MH892432.

**Table 1:** The genotypic and allelic frequencies of *POU1F1*-*Alu*I and *POU1F1*-*Pst*I analyses in Saanen dairy goats.

Loci	Farm	N		*POU1F1*			*POU1F1* allele		$\chi^{2}$
				genotypes			frequency		
				TT	TC	CC	T	C	
*POU1F1*-*Alu*I	Bornova	60	Obs.	36.7	38.3	25.0	0.56	0.44	3.20
			Exp.	31.9	49.7	19.3			
	Manisa	48	Obs.	77.1	22.9	–	0.88	0.12	0.72
			Exp.	78.3	20.5	1.2			
	Total	108	Obs.	54.6	31.5	13.9	0.70	0.30	6.76${}^{*}$
			Exp.	49.4	41.9	8.7			
*POU1F1*-*Pst*I	Bornova	60	Obs.	66.6	26.7	6.7	0.80	0.20	1.87
			Exp.	63.9	32.2	3.9			
	Manisa	48	Obs.	62.5	37.5	–	0.81	0.19	2.39
			Exp.	65.8	30.8	3.4			
	Total	108	Obs.	64.8	31.5	3.7	0.80	0.20	0.00
			Exp.	64.8	30.6	3.7			

Similar to our results, the TT and TC genotypes were observed in the *POU1F1*-*Alu*I locus in the Chinese indigenous breed, and the CC genotype was found to be very low in only two of the nine breeds (Lan et al., 2007b, 2009a). In *POU1F1*-*Pst*I locus, genotype frequencies of TT and TC were observed as 91.7 % and 8.3 % respectively while CC genotype was not observed in native Chinese goats (Lan et al., 2009b). Also, as with Daga et al. (2013), TT and TC genotypes were observed in Italian goats and the CC genotype was not found. Similarly, it has been reported that the T allele is predominant in Chinese and Italian goats. The T allele was found to be monomorphic in Indian Barbari goats (Sharma et al., 2013).

Hershberg and Petrov (2008) reported that the tendency of codon usage may be different between codons of the same amino acid in different species, and that the frequency of the population may be lower in some populations as the codons reduce gene expression levels. The lack of each genotype *POU1F1*-*Alu*I and *POU1F1-Pst*I CC, and the presence of the small number of TC genotypes in the Manisa population, suggests that they may be related to the codon usage tendency, although the trends of codon usage in this study were not calculated.

In this study, all the genotype distributions of *POU1F1*-*Pst*I are found in Hardy–Weinberg equilibrium (p>0.05) except for *POU1F1-Alu*I (p<0.05). This can be because of random selection which is a result of artificial insemination. The reason for the absence of the CC genotype in the Manisa farm can be explained by the low number of samples. However, in some of the studies on the *POU1F1*-*Alu*I loci in various goat breeds, it should be noted that the absence of the CC genotype is found at a very low frequency regardless of the number of samples (Daga et al., 2013; Lan et al., 2009a, b).

### 3.1 Associations between genetic variations of the *POU1F1* gene and production traits

Individual milk yields of the Bornova and Manisa goats were recorded monthly, and protein, fat and dry matter ratios were determined in milk samples. Lactation milk yield was calculated as 731.94±14.17 kg, dry matter ratio 12.18±0.08, fat ratio 4.23±0.07 ,and protein ratio 3.31±0.02 in both farms (data not shown in table).

The relationships of the genotypes with milk yield and components for *POU1F1*-*Alu*I and *POU1F1*-*Pst*I loci are shown in Table 2. When two farms were evaluated together, TC and CC genotypes of *POU1F1*-*Alu*I locus have higher milk yields (784.58 and 786.07 kg, respectively) than that of the TT genotype (p<0.05). According to Lan et al. (2007b), the sixth exon was associated with high milk yield and birth weight. Also, in previous studies it has been reported that g.102T>C and g.216T>C polymorphisms of *POU1F1* gene are associated with the production traits such as milk yield and birth weight (Lan et al., 2007b, c). The relationships between genotypes, milk fat, protein ratio, and dry matter ratio in both farms were not found to be significant. The relationships between genotypes and milk fat ratio were not found to be significant, but the CC genotype in the *POU1F1*-*Alu*I locus appeared to have the highest (4.44 %) milk fat ratio. The dry matter ratio of the TT genotype was found to be higher (12.87 %), which was statistically significant, than that of the TC genotype (12.24 %) in Manisa farm (p<0.05) for *POU1F1*-*Alu*I locus (data not shown in Table 2). The relationships between genotypes and milk components were not found significant for *POU1F1* / *Pst*I locus. Similar to our results, Zhou et al. (2016) reported that there was no significant relationship between *POU1F1*-*Pst*I and milk performance in Guanzhong dairy goats. But they found that the TT genotype was higher than other genotypes for milk fat content and average milk fat.

**Table 2:** Relationships of *Alu*I and *Pst*I polymorphisms of the *POU1F1* gene with milk yield and milk components in Saanen dairy goats.

Traits	Loci	TT	TC	CC	p value
Lactation milk	*Alu*I	687.85${}^{b}$	784.58${}^{a}$	786.07${}^{a}$	0.015
yield (kg)	*Pst*I	738.92	711.20	786.25	0.500
Milk fat ratio	*Alu*I	3.70	4.08	4.44	0.832
(%)	*Pst*I	4.21	4.33	3.77	0.861
Milk protein	*Alu*I	3.29	3.33	3.34	0.940
ratio (%)	*Pst*I	3.30	3.31	3.45	0.767
Milk dry matter	*Alu*I	12.42	11.96	11.72	0.358
ratio (%)	*Pst*I	12.11	12.38	11.67	0.391

${}^{a,b}$ Values with different superscripts within the same row differ significantly (p<0.05). n: 108 sample.

Relationships between the genotypes obtained from *POU1F1*-*Alu*I, *POU1F1*-*Pst*I and growth traits such as birth weight, weaning weight and litter size characteristics are shown in Table 3. The relationships between genotypes for *POU1F1*-*Alu*I locus and birth weight and weaning weight were not found to be statistically significant at the Bornova farm. For *POU1F1*-*Alu*I locus, the CC genotype was found higher in litter size than the TT and TC genotypes at the Bornova farm, whereas the CC genotype was not found at the Manisa Farm (p<0.05). When the two farms were evaluated together, the CC and TC genotypes were found higher in litter size than the TT genotype (p<0.01). In contrast with our results, Feng et al. (2012) reported that the litter size was higher in the case of the TT genotypes at C256T in exon 3 and G682T (A228S) in exon 6 of *POU1F1* gene. According to Sun (2007), variants of the *POU1F1* gene have significant influences on birth weight and weight at 1.5 years old on Liangshan sheep.

**Table 3:** Relationships between the genotypes of *POU1F1-Alu*I, *POU1F1-Pst*I loci and growth traits.

Traits	Loci	N	TT	TC	CC	p value
Birth weight	*Alu*I	88+	3.97	3.90	3.60	0.79
(kg)	*Pst*I		3.74${}^{b}$	3.89${}^{\mathrm{ab}}$	4.73${}^{a}$	0.035
Weaning	*Alu*I	88+	20.03	21.08	19.84	0.61
weight (kg)	*Pst*I		19.87	21.18	23.55	0.536
Litter size		75${}^{+}$	1.31${}^{b}$	1.47${}^{b}$	1.86${}^{a}$	0.03
(lamb)	*Alu*I	87${}^{++}$	1.51${}^{b}$	2.81${}^{a}$	–	0.00
		162	1.44${}^{b}$	1.91${}^{a}$	1.87${}^{a}$	0.00
		75${}^{+}$	1.6	1.37	1.25	0.69
	*Pst*I	87${}^{++}$	1.78	1.83	–	0.52
		162	1.7	1.59	1.25	0.32

${}^{a,b}$ Values with different superscripts within the same row differ significantly (p<0.05). ${}^{+}$ Bornova. ${}^{++}$ Manisa sample number.

Differences between the CC, TC and TT genotypes (4.73, 3.89, and 3.74 kg, respectively) were found to be statistically significant (p<0.05) with the *POU1F1*-*Pst*I locus for birth weight at the Bornova farm. For the *POU1F1*-*Alu*I locus, birth weight was determined highest with the TT genotype, although it was not found to be statistically significant.

According to Ma et al. (2017), DQ826397.1:g.102T>G (SNP1), DQ826397.1:g.279T>C (SNP2) and NC_019460.2:g.1100T>A (SNP6) were associated with some growth traits such as hucklebone width, body weight, chest width, and chest circumference. The SNP1 locus at exon 6 had a significant association with hucklebone width (p<0.05) and the hucklebone width index (p<0.05) in Guanzhong dairy goats.

These results indicate that the *POU1F1*-*Alu*I locus has significant effects on milk performance and litter size. Also the *POU1F1*-*Pst*I locus has a significant effect on birth weight. Therefore, the SNPs of *POU1F1* may be useful for potential marker-assisted selection in dairy goat breeding.

## 4 Conclusion

The *POU1F1* gene is a transcription factor gene that plays a role in the regulation of expression of genes such as *GH*, *PRL* and *TSH*β. It is also thought that *POU1F1* may be a potential candidate gene for the marker-assisted selection of production traits such as milk yield, components, growth, and reproduction. Following the determination of polymorphisms, the determination of the mRNA expression level of the *POU1F1* gene would be useful to reveal any indirect effects on the genes that could affect the quantitative traits.

## Supplement

10.5194/aab-62-249-2019-supplement The supplement related to this article is available online at: https://doi.org/10.5194/aab-62-249-2019-supplement.

## Data Availability

The sequences of the samples studied are provided in the supplement.
