# Supplementary material for: Associations between genetic variants of the POU1F1 gene and production traits in Saanen goats
Source: Arch Anim Breed. 2019 May 3;62(1):249–55. doi: 10.5194/aab-62-249-2019 (PMC6852870; doi:10.5194/aab-62-249-2019)
Supplement: The supplement related to this article is available online at: https://doi.org/10.5194/aab-62-249-2019-supplement. [file aab-62-249-supplement.pdf]

Supplement of Arch. Anim. Breed., 62, 249–255, 2019
https://doi.org/10.5194/aab-62-249-2019-supplement
© Author(s) 2019. This work is distributed under
the Creative Commons Attribution 4.0 License.

Open Access
Archives Animal Breeding

Supplement of

Associations between genetic variants of the *POU1F1* gene and production traits in Saanen goats

Raziye Işık and Güldehen Bilgen

Correspondence to: Raziye Işık (risik@nku.edu.tr)

The copyright of individual parts of the supplement might differ from the CC BY 4.0 License.

>Seq1 [organism=Capra hircus] Capra hircus POU1F1 (Pit-1) gene, exon 6 and partial cds

CCATCATCTCCCTTCTTCTTTCCTGCCAACTCCCCACCTCCCAGTATTGCTGCTAAAGACGCCCTGGAGAGACACTTT
GGAGAACAGAATAAGCCTTCCTCGCAGGAGATCCTGAGGATGGCTGAAGAACTAAACCTGGAGAAAGAAGTGGT
GAGGGTTTGGTTTTGTAACCGAAGACAGAGAGAAAAACGGGTGAAAACAAGCCTGAATCAGAGCTTATTTCCTAT
CTCTAAGGAGCATCTTGAATGCAGATAGGTCTCCCATTGTGTAATAGCGAGTTTTTCTGCTTTTCTTTCCCTTCTCTTC
TCCAGCCAAAGTAGAAATCAGTTATTTGGTTAGCTTCCAAACGTCACATCAGTAATGTTTGCAGAAGTGTTTCTCTT
CTACTTTAAAAACAAATACAATTTAAATTATGTTGATGAATTATTCTCAGAAGGCACATTGTACATT
